# tRNA-Derived Small Non-Coding RNAs as Novel Epigenetic Molecules Regulating Adipogenesis

**DOI:** 10.3390/biom9070274

**Published:** 2019-07-11

**Authors:** Linyuan Shen, Zhendong Tan, Mailin Gan, Qiang Li, Lei Chen, Lili Niu, Dongmei Jiang, Ye Zhao, Jinyong Wang, Xuewei Li, Shunhua Zhang, Li Zhu

**Affiliations:** 1 College of Animal Science and Technology, Sichuan Agricultural University, Chengdu 611130, China; 2 Farm Animal Genetic Resource Exploration and Innovation Key Laboratory of Sichuan Province, Sichuan Agricultural University, Chengdu 611130, China; 3 Sichuan Province General Station of Animal Husbandry, Chengdu 611130, China; 4 Chongqing Academy of Animal Science, Chongqing 402460, China

**Keywords:** tRFs, adipogenesis, 3T3-L1, KLF family, transcriptome

## Abstract

tRNA-derived fragments (tRFs), a novel type of non-coding RNA derived from tRNAs, play an important part in governing gene expressions at a post-transcriptional level. To date, the regulatory mechanism of tRFs governing fat deposition and adipogenesis is completely unknown. In this study, high fat diet was employed to induce an obese rat model, and tRFs transcriptome sequencing was conducted to identify differentially expressed tRFs that response to obesity. We found out that tRF^GluTTC^, which promoted preadipocyte proliferation by increasing expressions of cell cycle regulatory factors, had the highest fold change in the 296 differentially expressed tRFs. Moreover, tRF^GluTTC^ also suppressed preadipocyte differentiation by reducing triglyceride content and lipid accumulation, and by decreasing expressions of genes that related to fatty acid synthesis. According to results of luciferase activity analysis, tRF^GluTTC^ directly targeted Kruppel-like factor (KLF) 9, KLF11, and KLF12, thus significantly suppressing mRNA expressions of these target genes. Moreover, tRF^GluTTC^ suppressed adipogenesis, accompanying by suppressing expressions of adipogenic transcription factors (*aP2*, *PPARγ*, and *C/EBPα*). In conclusion, these results imply that tRF^GluTTC^ may act as a novel epigenetic molecule regulating adipogenesis and could provide a new strategy for the intervention treatment of obesity.

## 1. Introduction

Currently, epidemic obesity is seriously affecting humans’ health worldwide. Obesity is also associated with high occurrence risks of multiple diseases, such as cardiovascular disease, type II diabetes, hypertension, respiratory disease, and a variety of cancers [1]. Obesity can lead to adipose tissue mass increase, and also advance the size and number of adipocytes differentiated from preadipocytes at cellular level [2]. Therefore, to understand obesity and its associated diseases, exploring potential molecular mechanisms governing adipocyte differentiation is necessary. Actually, the complex process of preadipocyte differentiation is regulated by sequential activations of multifarious transcription factors, such as PPARγ (peroxisome proliferator-activated receptor gamma), C/EBPa (CCAAT enhancer binding protein alpha), and KLF (Kruppel-like factor) family [2,3,4]. However, our knowledge about transcription factors and molecular switches regulating adipocyte differentiation is far from complete.

In recent decades, non-coding RNAs are known as pivotal epigenetic molecules regulating almost all biological processes, including adipogenesis. Furthermore, many miRNAs, lncRNA, and circRNAs have been proved to govern adipogenesis tightly [5,6,7]. Recently, a novel type of small RNAs different from typical noncoding RNAs has been discovered. They were generated by non-random cleaving from mature tRNAs under a variety of stress conditions, such as myocardial hypertrophy and chronic hepatitis [8,9]. Therefore, these small RNAs are fragments derived from tRNA, and they are defined as tRFs. Subsequent studies have shown that tRFs have similar functions as miRNAs in regulating gene expressions at post-transcriptional level by banding argonaute proteins [10,11]. Therefore, we hypothesized that tRFs may be involved in the epigenetic regulatory network during preadipocyte differentiation.

To verify this hypothesis, deep tRFs sequencing was conducted to identify vital tRFs involved in fat deposition in vivo, and 3T3-L1 preadipocyte was employed to verify functions of tRFs on adipogenesis in vitro. These studies imply that tRFs may act as novel epigenetic molecules for intervention treatment of obesity and metabolic syndrome.

## 2. Materials and Methods 

In this study, animal care and all procedures were performed according to instructions of Institutional Animal Care and Use Committee of Sichuan Agricultural University, Sichuan, China, under permit No. DKY-B20131403 (Ministry of Science and Technology, China, approved on 15 June 2004). Sixteen 6-week-old male Strague Dawley (SD) rats were randomly divided into two groups: LFD (low fat diet, *n* = 8) and HFD (high fat diet, 40% fat, *n* = 8). After 100 days of feeding, a glucose tolerance test (GTT) was conducted on rats following our previous study [12]. Subsequently, adipose tissues and blood samples were sampled. HE staining was carried out to calculate areas of adipocytes. Detection kits (Nanjing Jiancheng Biology Engineering Institute, China) were used to detect concentration of serum cholesterol (TC) and triglyceride (TG). 

Six adipose tissue samples were sent to Annoroad Gene Technology Co. Ltd. (Beijing, China) for tRFs sequencing. Briefly, fractions of 15–40 nt were excised and purified from total RNA, and obtained small RNA fractions were used to construct tRFs sequencing library through ligating adapters and RT-PCR reaction. Raw datum files in FASTQ format were generated through Illumina Hi-Seq 2500 platform. The process of tRFs-seq datum analysis was carried out following a previous study [13]. All tRFs sequencing data were deposited at NCBI’s Gene Expression Omnibus under accession numbers of GSE129685.

3T3-L1 cells were proliferated and differentiated, and then transfected with mimics and inhibitors following steps in our previous study [4]. tRF^GluTTC^ mimics (5′- TCCCACATGGTCTAGCGGTTAGGATTCCTGGTTTT-3′) and specific chemically modified inhibitors (antisense oligonucleotide, 5′- AGGGTGTACCAGATCGCCAATCCTAAGGACCAAAA-3′) were designed and synthesized by RiboBio (Guangzhou, China), and then transfected into 3T3-L1 cells by mixing with Lipofectamine 2000 reagent (Invitrogen, Carlsbad, CA, USA). Briefly, 2 μL mimic (20 μM) or 4 μL inhibitor (20 μM) with lipid carrier (2:1, *v/v*) were subjected to transfection in 500 μL Opti-MEM medium for each well in standard 24-well plates. CCK-8 and EdU assays were conducted following operation instructions to survey the proliferation rate of 3T3-L1 after transfecting with tRF^GluTTC^ mimics or inhibitors. Differentiated 3T3-L1 cells, which were transfected with tRF^GluTTC^ mimics or inhibitors, were stained with Oil Red O (60% saturability), and triglyceride assay was operated using a detection kit (Catalogue No. TR22421, ThermoFisher Scientific, Madison, WI, USA). For the luciferase reporter analysis, modified psiCHECK™-2 vectors were constructed by inserting wild-type and mutant KLF family 3’-UTR into psiCHECK™-2, respectively. Then HeLa cells were cultured to detect luciferase activities after co-transfecting with modified psiCHECK™-2 vectors and tRF^GluTTC^ mimics, respectively. Dual-Glo Luciferase Assay System (Promega, Madison, WI, USA) was employed to test luciferase activities. tRFs and mRNAs quantification were performed according to our previous study [9]. All data were presented as mean ± standard deviation. Difference significance analysis was performed using SPSS software (version 23; SPSS Inc., Chicago, IL, USA) with the program of Student’s *t*-test, the level of statistical significance was set at *p* < 0.05. 

## 3. Results and Discussion

### 3.1. High Fat Diet Induces Obesity Phenotypes in the Rat Model

**Figure 1:**
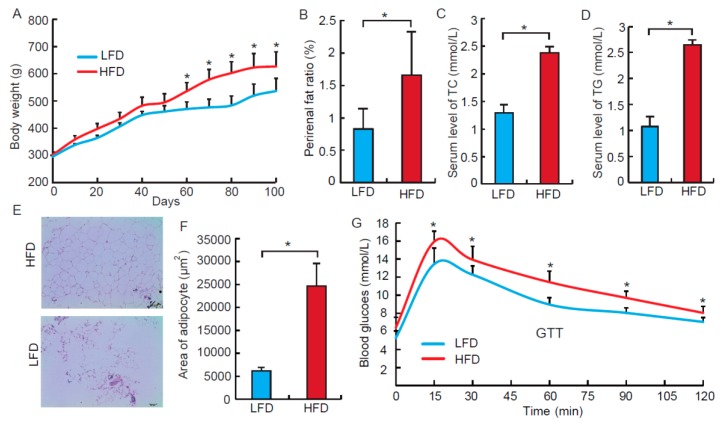
High fat diet induces obese rat model. (**A**) The body weight of Strague Dawley (SD) rats that were fed with normal chow and high fat diet, *n* = 8. (**B**) The ratio of perirenal fat to body weight in the low fat diet (LFD) and the high fat diet (HFD) groups, *n* = 8. (**C**,**D**) The concentration of serum cholesterol (TC) and triglyceride (TG) in the LFD and HFD group, *n* = 8. (**E**) HE staining of perirenal fat from normal and obese SD rats, *n* = 3. Scale bar = 100 μm. (**F**) The average adipocyte area of perirenal fat from normal and obese SD rats, *n* = 3. (**G**) The blood glucose concentration of rats after an intravenous glucose tolerance test (i.v. test of glucose tolerance (GTT)), *n* = 8. All data were presented as means ± standard deviation. * *p* < 0.05.

To investigate functions of tRFs on fat deposition and adipogenesis, SD rats were employed to construct the obesity model. As shown in Figure 1A–D, after 100 days of feeding, the HFD group had a higher body weight, a higher perirenal fat ratio, and higher total TC and TG in serum comparing with the LFD group (*p* < 0.05), which were consistent with previous studies reported about the prototypical phenotypes of an obese model [12]. Additionally, the mean area of adipocytes in the HFD group was higher than that of the LFD group by four times (*p* < 0.05) (Figure 1E,F). Furthermore, glucose tolerance is an accompanying clinical symptom of an obese model [14]. As expected, test of glucose tolerance (GTT) indicated that obese rats resulted in a severe insulin resistance symptom (Figure 1G, *p* < 0.05). Therefore, considering these findings, we believe that the obese model used in this study was successful.

### 3.2. tRFs Profile Influenced by the Stress of Obesity

**Figure 2:**
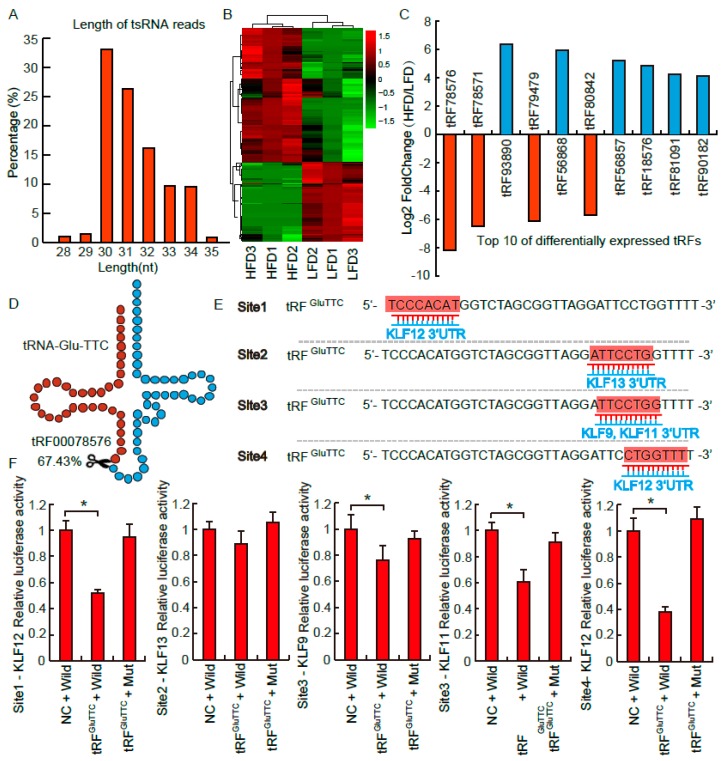
tRNA derived fragments (tRFs) transcriptome sequencing and target gene prediction. (**A**) The average length of identified tRFs from 6 libraries. (**B**) Heat map diagram of differentially expressed tRFs between normal and obese rats. (**C**) The list of top 10 differentially expressed tRFs between normal and obese rats. (**D**) tRF78576 cleaved from tRNA-Glu-TTC, which occupied 67.43% of fragments derived from tRNA-Glu-TTC (**E**) tRF^GluTTC^ has complementary sequences with 3’UTR of Kruppel-like factor (KLF) 12, KLF12, KLF9, and KLF11. (**F**) The inhibitory effect of tRF^GluTTC^ on target genes measured by luciferase assays. All data were presented as means ± standard deviation. * *p* < 0.05.

To explore the differences of tRFs profile in adipose in response to the stress of obesity, perirenal fat sampled from the HFD and LFD groups was performed with tRFs transcriptome sequencing (15–40 nt). As shown in Figure 2A, we found the length of tRFs mainly enrich in 30–34 nt, which was highly consistent with previous findings [13,15]. In our research, 296 differentially expressed tRFs were identified between the HFD and LDF groups, among which 170 tRFs were highly expressed in HFD, and 126 tRFs were highly expressed in LFD (Figure 2B). As shown, among the listed top 10 differentially expressed tRFs, the lowest fold change of tRFs was 15 times, and the highest fold change was more than 250 times (Figure 2C). Therefore, huge variations of tRFs profile between the HFD and LFD groups suggested that tRFs might have potential regulatory functions in fat deposition in vivo.

### 3.3. tRF^GluTTC^ Directly Targets KLF Family

Recently, a number of evidences proved that tRFs had similar functions as miRNAs by guiding Ago2 to regulate target gene expressions [16]. However, there were some dissimilar molecular mechanisms between tRFs and miRNAs. For example, tRFs-targeting sites were not only located in 3’UTR, but also located in 5’UTRs and CDSs [11]. Furthermore, the complementary sequences of tRFs were not always located in 5’ tail end (2–8 sites) [9]. Here, we hypothesized that tRFs might have target genes related to adipogenesis. Firstly, the top one differentially expressed tRFs was selected to explore its target genes. As shown in Figure 2D, tRF78576 was cleaved from mature tRNA-Glu-TTC, which occupied for 67.43% fragments derived from tRNA-Glu-TTC and named as tRF^GluTTC^ in this study. These results were consistent with previous studies reporting that tRNA-derived tRFs were not random products of tRNA fragments, but possessed tissue-specific characteristics [17]. The results of precise cleaving modulations of tRNA-Glu-TTC also implied that it had a potential role in regulating adipogenesis. According to bioinformatics analyses, tRF^GluTTC^ has 7-mer complementary sequences with KLF family, such as KLF9, KLF11, KLF12, and KLF13. Previous studies have reported that transcription factors from KLF family were important regulators of adipogenesis [18,19,20]. For example, KLF9 was identified as a key pro-adipogenic transcription factor through up-regulating the expression of PPARγ and C/EBPα [21]. To further verify that KLF family were direct target genes of tRF^GluTTC^, a double fluorescent reporter analysis was carried out. As shown in Figure 2F, HeLa cells co-transfected with tRF^GluTTC^ mimics and WT-KLF12/WT-KLF9/WT-KLF11 could significantly decrease the relative luciferase activity (*p* < 0.05). However, decreasing of luciferase activity was not observed in HeLa cells co-transfected with tRF^GluTTC^ mimics and mutant-KLF13 luciferase plasmids. To sum up, these data indicate that tRF^GluTTC^ targeting KLF family might be one of the potential epigenetic regulatory pathways in governing adipogenesis. 

### 3.4. tRF^GluTTC^ Regulates Proliferation and Differentiation of Preadipocytes

**Figure 3:**
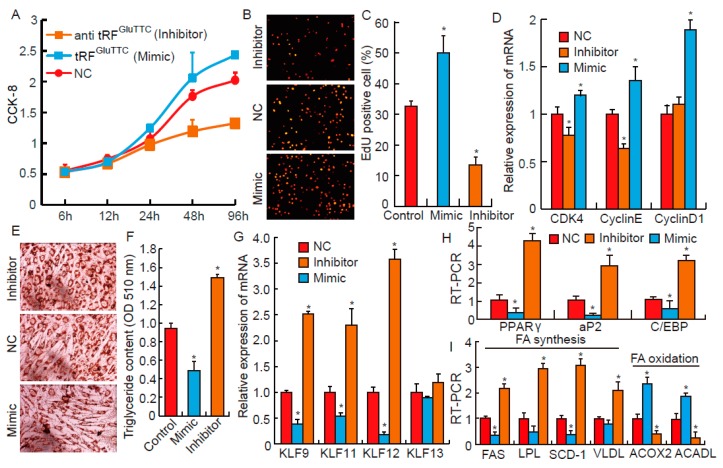
The role of tRF^GluTTC^ in proliferation and differentiation of preadipocytes. (**A**) The proliferation ability of 3T3-L1 preadipocyte was measured by CCK-8. (**B**,**C**) 3T3-L1 preadipocyte proliferation was measured by EdU staining. (**D**) The relative mRNA expression levels of cell cycle regulatory factors (CDK4, Cyclin E, and Cyclin D1) by RT-PCR. (**E**,**F**) Oil Red O staining and triglyceride content of terminally differentiated (Day 8) adipocytes that transfected with tRF^GluTTC^ mimics and inhibitors. (**G**) The relative expression of KLF family (KLF9, KLF11, KLF12, and KLF13) by RT-PCR. (**H**) The relative expression of adipogenic transcription factors (*aP2*, peroxisome proliferator-activated receptor gamma (*PPARγ*), and CCAAT enhancer binding protein alpha (*C/EBPα*)) by RT-PCR. (**I**) The relative expression of genes related to fatty acid synthesis and oxidation by RT-PCR. All data were presented as means ± standard deviation. * *p* < 0.05.

Previous studies have reported that tRFs derived from tRNA-Glu-TTC were closely related with moyamoya disease and triple negative breast cancer [22,23]. However, we speculated that tRF^GluTTC^ may play other important roles in regulating adipogenesis based on the fact that tRF^GluTTC^ directly targets KLF family genes. In this study, 3T3-L1 preadipocytes, a widely used adipocyte differentiation model, were used to further explore the pathways of tRF^GluTTC^ involved in adipogenesis. Based on the results of CCK-8 assay, we found out that the proliferation rate of 3T3-L1 preadipocytes increased when tRF^GluTTC^ overexpressed, but it decreased when the expression of tRF^GluTTC^ decreased (Figure 3A). These results were consistent with EdU staining. As shown in Figure 2B,C, overexpression or knockdown of tRF^GluTTC^ could significantly increase or decrease the amount of EdU-positive cells, respectively. Additionally, to further verify that tRF^GluTTC^ has the function of promoting preadipocyte proliferation, mRNA expressions of cell cycle regulatory factors were also detected. For example, Cyclin D1, CDK4 (cyclin-dependent kinases), and Cyclin E were essential factors for maintaining G1/S phase transition in mammalian cells [24,25]. As illustrated in Figure 3D, increased expression levels of Cyclin D1, CDK4, and Cyclin E were observed in tRF^GluTTC^ mimic group, while knockdown of tRF^GluTTC^ could significantly decrease CDK4 and Cyclin E expression. Thus, all data together indicate that tRF^GluTTC^ could promote 3T3-L1 preadipocyte proliferation. 

Lipid accumulation in adipose tissues is on the basis of adipocyte proliferation and differentiation. To further identify the function of tRF^GluTTC^ in adipogenesis, the differentiation extent of 3T3-L1 cells that transfected with tRF^GluTTC^ mimics or inhibitors were measured. After eight days of differentiation, according to Oil Red O staining results, overexpression or knockdown of tRF^GluTTC^ significantly suppressed or promoted 3T3-L1 differentiation, respectively (Figure 3E). These results were highly consistent with triglycerides (TG) accumulation in 3T3-L1 preadipocytes (Figure 3F). Moreover, we found out that transfecting tRF^GluTT^ mimics or inhibitors into 3T3-L1 could significantly suppress or promote mRNA expressions of KLF9, KLF11, and KLF12, respectively (Figure 3G), which were in accordance with tRF^GluTTC^ directly targeting KLF9, KLF11, and KLF12. *aP2, C/EBPα*, and *PPARγ* were essential transcription factors for regulating adipogenesis [26], and all expressions were down-regulated in the mimic group. However, opposite results were observed in the inhibitor group (Figure 3H). Moreover, both decreased fatty acid oxidation and increased fatty acid synthesis can improve lipid accumulation in adipocytes. As expected, we found that overexpression of tRF^GluTTC^ could significantly decrease and increase expressions of genes related to fatty acid oxidation and fatty acid synthesis, respectively. On the contrary, knockdown of tRF^GluTTC^ had opposite results (Figure 3I). The expression pattern of these genes was consistent with previous studies reporting about phenotypes of fatty acid metabolism [4,12]. In all, these results imply that tRF^GluTTC^ could suppress 3T3-L1 preadipocyte differentiation.

## 4. Conclusions

In summary, our study firstly investigated variations of tRFs profile in response to the stress of obesity. A total of 296 differentially expressed tRFs were found between normal and obese adipose tissues. Interestingly, tRF^GluTTC^ had the highest fold change of tRFs, which could directly target KLF family, such as KLF9, KLF11, and KLF12. Overexpression of tRF^GluTTC^ significantly promoted 3T3-L1 preadipocyte proliferation, but significantly suppressed 3T3-L1 differentiation. Thus, these findings imply that tRFs may act as novel epigenetic molecules for governing adipogenesis.
